# BRAF v600E–mutant cancers treated with vemurafenib alone or in combination with everolimus, sorafenib, or crizotinib or with paclitaxel and carboplatin (VEM-PLUS) study

**DOI:** 10.1038/s41698-022-00341-0

**Published:** 2023-02-18

**Authors:** Blessie Elizabeth Nelson, Jason Roszik, Filip Janku, David S. Hong, Shumei Kato, Aung Naing, Sarina Piha-Paul, Siqing Fu, Apostolia Tsimberidou, Maria Cabanillas, Naifa Lamki Busaidy, Milind Javle, Lauren Averett Byers, John V. Heymach, Funda Meric-Bernstam, Vivek Subbiah

**Affiliations:** 1 Departments of Investigational Cancer Therapeutics, The University of Texas MD Anderson Cancer Center, Houston, TX USA; 2 Melanoma Medical Oncology, The University of Texas MD Anderson Cancer Center, Houston, TX USA; 3 Endocrine Neoplasia and Hormonal Disorders, The University of Texas MD Anderson Cancer Center, Houston, TX USA; 4 Gastrointestinal Medical Oncology, The University of Texas MD Anderson Cancer Center, Houston, TX USA; 5 Thoracic/Head and Neck Medical Oncology, The University of Texas MD Anderson Cancer Center, Houston, TX USA

**Keywords:** Targeted therapies, Drug development, Cancer genomics

## Abstract

Combined BRAF + MEK inhibition is FDA approved for BRAF V600E-mutant solid tumors except for colorectal cancer. However, beyond MAPK mediated resistance several other mechanisms of resistance such as activation of CRAF, ARAF, MET, P13K/AKT/mTOR pathway exist among other complex pathways. In the VEM-PLUS study, we performed a pooled analysis of four phase one studies evaluating the safety and efficacy of vemurafenib monotherapy and vemurafenib combined with targeted therapies (sorafenib, crizotinib, or everolimus) or carboplatin plus paclitaxel in advanced solid tumors harboring *BRAF V600* mutations. When vemurafenib monotherapy was compared with the combination regimens, no significant differences in OS or PFS durations were noted, except for inferior OS in the vemurafenib and paclitaxel and carboplatin trial (*P* = 0.011; HR, 2.4; 95% CI, 1.22–4.7) and in crossover patients (*P* = 0.0025; HR, 2.089; 95% CI, 1.2–3.4). Patients naïve to prior BRAF inhibitors had statistically significantly improved OS at 12.6 months compared to 10.4 months in the BRAF therapy refractory group (*P* = 0.024; HR, 1.69; 95% CI 1.07–2.68). The median PFS was statistically significant between both groups, with 7 months in the BRAF therapy naïve group compared to 4.7 months in the BRAF therapy refractory group (*P* = 0.016; HR, 1.80; 95% CI 1.11–2.91). The confirmed ORR in the vemurafenib monotherapy trial (28%) was higher than that in the combination trials. Our findings suggest that, compared with vemurafenib monotherapy, combinations of vemurafenib with cytotoxic chemotherapy or with RAF- or mTOR-targeting agents do not significantly extend the OS or PFS of patients who have solid tumors with BRAF V600E mutations. Gaining a better understanding of the molecular mechanisms of BRAF inhibitor resistance, balancing toxicity and efficacy with novel trial designs are warranted.

## Introduction

*BRAF* mutations are seen in up to 10% of various cancers, including melanoma (40–60%), thyroid cancer (50%), glioma (11%), colorectal carcinoma (10%), cholangiocarcinoma (6%), non-small cell lung carcinoma (NSCLC) (3%), hairy cell leukemia, multiple myeloma, and Langerhans cell histiocytosis^1^. The B-Raf proto-oncogene, *BRAF*, activates cytoplasmic serine and threonine kinase and stimulates mitogen-activated protein kinase (*MAPK/MEK*) signaling^2^.

For patients with pathognomonic V600E mutations, which are located on codon 600 of exon 15 in multiple neoplasms, several drugs are available such as dabrafenib plus trametinib, vemurafenib or encorafenib monotherapy, Vemurafenib plus cobimetinib and Encorafenib plus binimetinib^3^. Soon after the development of BRAF inhibitors it was realized that there was a complete recovery of the MAPK pathway and additional MEK inhibition was needed in combination to overcome this mechanism. Rapid clinical translation and registrational trials have led to US FDA approval of three BRAFi/MEKi combinations dabrafenib plus trametinib (melanoma, NSCLC, anaplastic thyroid cancer (ATC), and BRAF + solid tumors), vemurafenib plus cobimetinib (metastatic melanoma), and encorafenib plus binimetinib (approved in metastatic melanoma)^4–7^.

However, beyond MAPK pathway as a resistance mechanism, several other complex resistance mechanisms exist, such as activation of *CRAF, ARAF, MET*, P13K/AKT/ *mTOR* pathway and other complex mechanisms^8^. RAF inhibition combined with co-targeting some of these targets with sorafenib (*CRAF, CD117*, vascular endothelial growth factor receptors 1–3; *PDGFRβ*; FMS-like tyrosine kinase 3; *KIT* and rearranged during transfection proto-oncogene receptor tyrosine kinases), Crizotinib (*MET*, anaplastic lymphoma kinase (*ALK*), and *ROS* proto-oncogene 1), everolimus (mTOR1 inhibitor) and chemotherapy (nonspecific complex resistance mechanisms) have been explored^9–12^.

In this VEM + PLUS study, we sought to analyze the clinical outcomes of patients previously treated with these agents when combined with BRAF inhibitor vemurafenib from previously published four phase 1 trials (NCT01524978, NCT01596140, NCT01636622, and NCT01531361)^13–16^. We performed a pooled analysis evaluating the safety and efficacy of vemurafenib monotherapy and vemurafenib combined with various combinations of targeted therapies (sorafenib, crizotinib, or everolimus) or standard chemotherapy (carboplatin plus paclitaxel) in patients with advanced and refractory solid tumors harboring *BRAF* V600 mutations.

## Results

### Patient characteristics

Patient and disease characteristics are given in Table 1. Ninety-nine patients (57 males and 42 females) with *BRAF* mutant solid tumors were enrolled across four phase I clinical trials (NCT01524978, NCT01596140, NCT01636622, and NCT01531361) between January 2012 and October 2020. Twenty-eight patients were enrolled in a trial of vemurafenib monotherapy (V; NCT01524978), 18 patients in a trial of vemurafenib with paclitaxel and carboplatin (V + P + C; NCT01636622), 18 patients in a trial of vemurafenib and everolimus (V + E; NCT01596140), and 35 patients in a trial of vemurafenib with crizotinib (V + C; n = 14) or sorafenib (V + S; *n* = 21; NCT01531361)^17–20^. We analyzed the effects of V + C and V + S separately. The median patient age was 57 years (range,10–79 years). Seventy-eight percent of patients had an Eastern Cooperative Oncology Group performance status of 1. Tumor types were cutaneous melanoma (36%), NSCLC (13%), thyroid cancer (12%), low- and high-grade gliomas (7%), colorectal cancer (6%), cholangiocarcinoma (5%), Erdheim-Chester disease (3%), and others (18%), which included neuroendocrine, ovarian, and pancreatic cancers and primary carcinomas of unknown origin). Forty-one patients (41%) had received prior BRAF inhibitors and 58 patients (59%) had not. Most patients (67%) had received at least two lines of prior therapies. Thirty-six patients (36%) crossed over to one of the four phase 1 trials at the time of disease progression with further 1–2 trial participations. At data cutoff, six patients (6%) were alive (one patient in the V trial, two in the V + C arm, one in the V + E trial, and two in the V + S arm).

**Table 1:** Baseline patient characteristics.

Patient Characteristics	All Patients (*N* = 99)	Vemurafenib (*n* = 28)	Vemurafenib + Paclitaxel + Carboplatin (*n* = 18)	Vemurafenib + Crizotinib (*n* = 14)	Vemurafenib + Everolimus (*n* = 18)	Vemurafenib + Sorafenib (*n* = 21)
Age, median (range)	57 (10–79)	60.5 (24–77)	55 (38–75)	55 (39–76)	59 (10–79)	54 (24–76)
Gender, *n* (%)						
Male	57 (58%)	16 (57%)	10 (56%)	7 (50%)	12 (67%)	12 (57%)
Female	42 (42%)	12 (43%)	8 (44%)	7 (50%)	6 (33%)	9 (43%)
ECOG, *n* (%)						
0	11 (11%)	4 (14%)	0	0	6 (33%)	1 (5%)
1	77 (78%)	23 (82%)	16 (89%)	12 (86%)	9 (50%)	17 (81%)
2	10 (11%)	1 (4%)	2 (11%)	2 (14%)	3 (17%)	2 (10%)
Primary tumor, *n* (%)						
Neuroendocrine	3 (3%)	1 (4%)	0	1 (7%)	0	1 (5%)
Colorectal	6 (6%)	2 (7%)	0	1 (7%)	2 (11%)	1 (5%)
Cholangiocarcinoma	5 (5%)	3 (11%)	1 (6%)	1 (7%)	0	0
Salivary gland	3 (3%)	2 (7%)	0	0	0	1 (5%)
Thyroid						
Anaplastic thyroid	3 (3%)	2 (7%)	0	0	1 (6%)	0
Papillary thyroid	9 (9%)	0	1 (6%)	1 (7%)	3 (17%)	4 (19%)
Erdheim-Chester disease	3 (3%)	3 (11%)	0	0	0	0
CUP	4 (4%)	2 (7%)	1 (6%)	0	1 (6%)	0
NSCLC	13 (13%)	10 (36%)	0	1 (7%)	1 (6%)	1 (5%)
CNS						
GBM	4 (4%)	2 (7%)	0	0	2 (11%)	0
Gliosarcoma	1 (1%)	1 (4%)	0	0	0	0
Anaplastic astrocytoma	2 (2%)	0	0	0	1 (6%)	1 (5%)
Pancreatic cancer	1 (1%)	0	1 (6%)	0	0	0
Cutaneous melanoma	36 (36%)	0	13 (72%)	9 (64%)	6 (33%)	8 (38%)
Ovarian serous carcinoma	3 (3%)	0	0	0	0	3 (14%)
Seminoma	1 (1%)	0	0	0	0	1 (5%)
Histiocytoma	1 (1%)	0	1 (6%)	0	0	0
Optic nerve glioma	1 (1%)	0	0	0	1 (6%)	0
Prior lines of systemic therapies, *n* (%)						
0–1	32 (32%)	15 (54%)	3 (17%)	3 (21%)	6 (33%)	5 (24%)
2–3	44 (44%)	12 (43%)	8 (44%)	5 (36%)	8 (44%)	11 (53%)
4–5	17 (17%)	1 (4%)	5 (28%)	3 (21%)	4 (22%)	4 (19%)
> 5	6 (6%)	0	2 (11%)	3 (21%)	0	1 (5%)
Patient status						
Alive	6 (6%)	1 (4%)	0	2 (14%)	1 (6%)	2 (10%)
Dead	80 (81%)	19 (68%)	16 (57%)	12 (86%)	14 (78%)	19 (90%)
Unknown	13 (13%)	8 (29%)	2 (11%)	0	3 (17%)	0
Median number of cycles, *n*	4	5	4.5	2	3	4
Crossover patients, *n*	36	5	10	14	5	11

*ECOG* Eastern Cooperative Oncology Group, *CUP* carcinoma of unknown primary site, *NSCLC* non-small cell lung cancer, *GBM* glioblastoma.

### Safety and tolerability

All patients included in the analysis received at least one treatment as part of the trial participation.

All patients experienced one or more treatment-related adverse events (Table 2). Overall, nine patients experienced dose-limiting toxicity (DLT), and seven patients withdrew consent because of toxicities. Eight patients withdrew consent for other reasons.

**Table 2:** Adverse events reported across the VEM-PLUS study.

Treatment-related adverse events	Vemurafenib monotherapy (*n* = 28)			Vemurafenib + Paclitaxel + Carboplatin (*n* = 18)			Vemurafenib + Crizotinib (*n* = 14)			Vemurafenib + Everolimus (*n* = 18)			Vemurafenib + Sorafenib (*n* = 21)		
	G1	G2	G3/G4	G1	G2	G3/G4	G1	G2	G3/G4	G1	G2	G3/G4	G1	G2	G3/G4
Neutropenia	0%	0%	0%	6%	22%	28%	0%	0%	0%	0%	0%	0%	0%	0%	0%
Anemia	7%	0%	0%	6%	28%	11%	0%	0%	14%	0%	0%	0%	0%	0%	0%
Leukopenia	0%	0%	0%	22%	33%	28%	0%	0%	0%	0%	0%	0%	0%	0%	0%
Thrombocytopenia	0%	0%	0%	17%	6%	22%	0%	0%	0%	0%	0%	6%	0%	0%	0%
Nausea	0%	0%	0%	28%	11%	6%	14%	0%	0%	22%	0%	0%	10%	0%	0%
Vomiting	0%	0%	0%	28%	11%	6%	0%	0%	0%	0%	0%	0%	5%	0%	0%
Fatigue	25%	11%	7%	39%	22%	28%	29%	21%	0%	28%	22%	22%	29%	0%	0%
Hypertension	4%	0%	0%	0%	0%	0%	0%	0%	0%	0%	0%	0%	0%	0%	10%
SCC	0%	0%	14%	0%	0%	0%	0%	0%	0%	0%	0%	0%	0%	0%	0%
Low lymphocyte count	0%	0%	4%	0%	0%	0%	0%	0%	0%	0%	0%	0%	0%	0%	0%
Retinal detachment	0%	0%	0%	0%	0%	0%	0%	0%	7%	0%	0%	0%	0%	0%	0%
Increased creatinine level	0%	0%	4%	17%	6%	0%	7%	7%	0%	6%	0%	0%	10%	0%	0%
Peripheral neuropathy	11%	7%	4%	33%	11%	0%	0%	0%	0%	11%	0%	0%	5%	0%	0%
Anorexia	11%	4%	0%	22%	17%	6%	7%	29%	0%	6%	17%	6%	0%	0%	0%
Diarrhea	0%	0%	0%	22%	6%	0%	0%	0%	0%	22%	6%	0%	24%	0%	5%
Mucositis	0%	0%	4%	11%	0%	6%	0%	0%	0%	33%	11%	0%	10%	0%	0%
Hyperglycemia	0%	0%	4%	0%	0%	0%	0%	0%	0%	0%	0%	0%	0%	0%	0%
Skin rash	29%	18%	7%	6%	0%	0%	14%	7%	14%	39%	17%	22%	14%	10%	0%
Keratoacanthoma	0%	0%	4%	0%	0%	0%	0%	0%	0%	0%	0%	0%	0%	0%	0%
Sweet syndrome	0%	0%	4%	0%	0%	0%	0%	0%	0%	0%	0%	0%	0%	0%	0%
Infection	0%	0%	7%	6%	0%	6%	0%	0%	0%	0%	0%	0%	0%	0%	0%
HFS	4%	4%	0%	0%	0%	6%	0%	0%	0%	0%	0%	0%	10%	5%	5%
Febrile neutropenia	0%	0%	0%	0%	0%	6%	0%	0%	0%	0%	0%	0%	0%	0%	0%
AST elevation	0%	0%	0%	11%	0%	6%	0%	0%	0%	11%	0%	0%	0%	0%	0%
Increased bilirubin level	0%	4%	0%	17%	11%	0%	0%	0%	0%	6%	0%	0%	0%	5%	0%
Alopecia	4%	0%	0%	44%	6%	0%	0%	0%	0%	0%	0%	0%	0%	0%	0%
ALT elevation	0%	0%	0%	6%	6%	0%	0%	0%	0%	6%	0%	0%	0%	0%	0%
Hypomagnesemia	0%	0%	0%	11%	0%	0%	7%	0%	0%	0%	0%	0%	0%	0%	0%
Myalgia	11%	18%	0%	6%	0%	0%	0%	0%	0%	17%	0%	0%	0%	0%	0%
Constipation	0%	0%	0%	17%	0%	0%	0%	7%	0%	0%	0%	0%	0%	0%	0%
Hypokalemia	0%	0%	0%	17%	0%	0%	0%	0%	0%	0%	0%	0%	0%	0%	0%
Hypoalbuminemia	0%	0%	0%	0%	6%	0%	0%	0%	0%	0%	0%	0%	0%	0%	0%
Headache	0%	0%	0%	6%	6%	0%	0%	0%	0%	0%	0%	0%	0%	0%	0%
Conjunctivitis	0%	0%	0%	6%	0%	0%	0%	0%	0%	0%	0%	0%	0%	0%	0%
Hypocalcemia	0%	0%	0%	6%	6%	0%	0%	0%	0%	0%	0%	0%	0%	0%	0%
Dysgeusia	0%	0%	0%	6%	0%	0%	0%	7%	0%	11%	0%	0%	0%	0%	0%
Dyspnea	0%	0%	0%	6%	0%	0%	0%	0%	0%	0%	0%	0%	0%	0%	0%
Dehydration	0%	0%	0%	0%	6%	0%	0%	0%	0%	0%	0%	0%	0%	0%	0%
Photosensitivity	7%	0%	0%	11%	11%	0%	0%	0%	0%	0%	0%	0%	0%	0%	0%
Blurry vision	0%	0%	0%	0%	0%	0%	0%	7%	0%	0%	6%	0%	0%	0%	0%
ALP Elevation	0%	0%	0%	0%	0%	0%	0%	0%	0%	6%	0%	0%	0%	0%	0%
Hypercholesterolemia	0%	0%	0%	0%	0%	0%	0%	0%	0%	22%	11%	0%	0%	0%	0%
Uveitis	0%	7%	0%	0%	0%	0%	0%	0%	0%	0%	0%	0%	0%	0%	0%
G5 adverse event	0%	0%	0%	0%	0%	6%	0%	0%	0%	0%	0%	0%	0%	0%	0%

*G* grade, *SCC* squamous cell carcinoma, *HFS* hand-foot syndrome, *AST* aspartate aminotransferase, *ALT* alanine transaminase, *ALP* alkaline phosphatase.

Twenty-eight patients in the V trial received a median of four cycles of therapy (range, 1–62 cycles). Thirty-six percent of patients experienced grade (G) 1 or G2 fatigue and 7% of patients developed G3 or G4 fatigue. Fourteen percent of patients developed stage 3 or 4 cutaneous squamous cell carcinoma. Seven percent developed G3 or G4 skin rashes. Four percent of patients developed G3 or G4 serum creatinine levels, peripheral neuropathy, mucositis, or sweet syndrome. Twenty-nine percent of patients developed G1 skin rashes and 18% developed G2 skin rashes. Eleven and eighteen percent of patients developed G1 or G2 myalgias, respectively. Notably, 50% of patients required dose reductions, primarily because of skin rashes and fatigue. One patient withdrew from the trial because of toxicity from nausea and vomiting, although no G5 or DLT was observed.

Eighteen patients in the V + P + C trial received a median of 4.5 cycles of therapy, and most patients received between 1 and 30 cycles. The predominant G3 or G4 events were neutropenia (28%), leukopenia (28%), fatigue (28%), thrombocytopenia (22%), and anemia (11%). The most common G1 events were alopecia (44%), fatigue (39%), peripheral neuropathy (33%), leukopenia (33%), and vomiting (28%), while anemia (28%) was the most common G2 event. One patient died from G4 thrombocytopenia that caused an intracranial hemorrhage. Seven patients required dose reductions because of thrombocytopenia, neutropenia, peripheral neuropathy, or hand-foot syndrome (HFS). Two patients withdrew from the trial because of G3 fatigue and G3 thrombocytopenia, and one patient withdrew secondary to financial toxicity.

Fourteen patients in the V + C arm received a median of two cycles of therapy, and most patients received between 1 and 14 cycles. Twenty-nine percent of patients developed G1 fatigue, 29% developed G2 anorexia, and 14% developed skin rashes. G3 and four adverse events included anemia and skin rashes. Five patients required dose reductions, predominantly due to G3 HFS, skin rashes, and fatigue.

Eighteen patients in the V + E trial, patients received a median of four cycles of therapy (range, 1–46 cycles). The predominant G1 and G2 adverse events were skin rashes (39%), mucositis (33%), and fatigue (28%). The predominant G3 and G4 events included fatigue (22%) and skin rashes (22%). Six patients required dose reductions for G3 skin rash and/or fatigue. Four patients withdrew from the trial because of G3 photosensitivity, G3 skin rash, and G3 fatigue.

In the V + S arm, 21 patients received a median of four cycles of therapy (range, 1–31 cycles). Six patients required dose reductions because of G3 diarrhea and skin rashes. The most common G1 and G2 adverse events were fatigue (29%) and diarrhea (24%), whereas the predominant G3 and G4 adverse events were hypertension (10%), diarrhea (5%), and HFS (5%).

### Antitumor activity

**Fig. 1:**
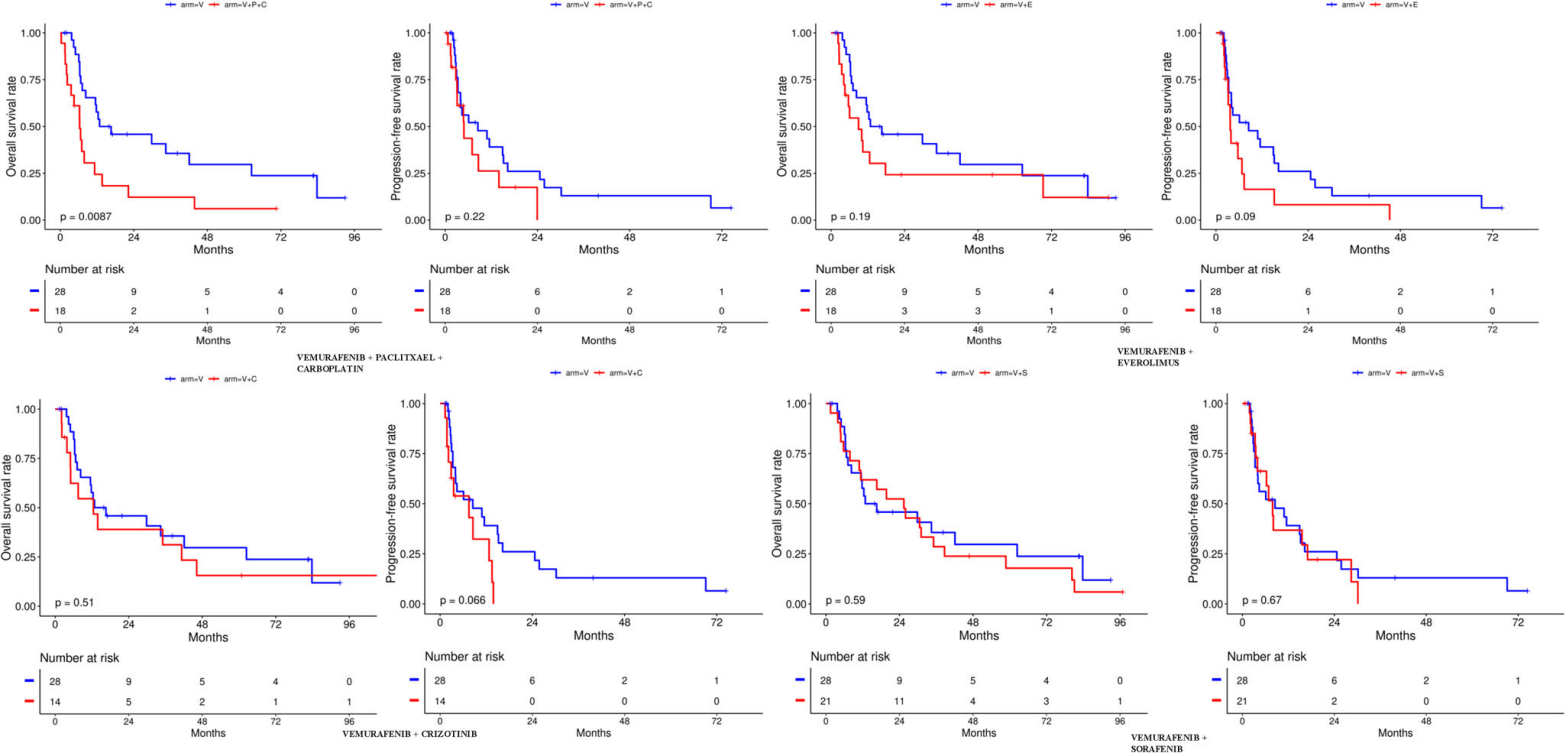
Best responses stratified by histology and duration of responses in the VEM-PLUS study. GBM, glioblastoma multiforme, NET neuroendocrine tumor, NSCLC non-small cell lung cancer, SCC squamous cell carcinoma, V vemurafenib. V + P + C vemurafenib + paclitaxel + carboplatin. V + E vemurafenib + everolimus. V + C vemurafenib + crizotinib.

Eighty-nine patients were evaluable for response (Table 3). The best responses are stratified by tumor histology and response duration in the swimmer plots in Fig. 1. Overall, two patients (2%) had confirmed complete responses (CRs) and 17 (19%) had confirmed partial responses (PRs), resulting in a confirmed ORR of 21%. Twenty-one patients (24%) had stable disease (SD) for more than 2 months. Therefore, the disease control rate (CR + PR + SD) was 45%.

**Table 3:** Tumor responses across the VEM-PLUS study.

Tumor responses	Total cohort (*n* = 89)	Confirmed responses (*n* = 19)	Vemurafenib monotherapy (*n* = 25)	Confirmed responses (*n* = 7)	Vemurafenib + Paclitaxel + Carboplatin (*n* = 15)	Confirmed responses (*n* = 3)	Vemurafenib + Crizotinib (*n* = 13)	Confirmed responses (*n* = 2)	Vemurafenib + Everolimus (*n* = 18)	Confirmed responses (*n* = 4)	Vemurafenib + Sorafenib(*n* = 18)	Confirmed responses (*n* = 3)
CR	2 (2.2%)	2 (2.2%)	1 (4%)	1 (4%)	1 (7%)	1 (6.6%)	0	0	0	0	0	0
PR	24 (27%)	17 (19%)	8 (32%)	6 (24%)	3 (20%)	2 (13%)	4 (31%)	2 (23%)	5 (28%)	4 (22%)	4 (22%)	3 (16.6%)
ORR	26 (29%)	19 (21%)	9 (36%)	7 (28%)	4 (27%)	3 (20%)	4 (31%)	2 (15%)	5 (28%)	4 (22%)	4 (22%)	3 (16.6%)
DCR: CR + PR + SD	67 (75%)	40 (45%)	19 (76%)	13 (52%)	13 (87%)	8 (53%)	8 (61.5%)	4 (31%)	14 (78%)	7 (39%)	13 (72%)	8 (44%)
PD	22 (25%)	N/A	6 (24%)	N/A	2 (13%)	N/A	5 (38%)	N/A	4 (22%)	N/A	5 (28%)	N/A
SD*	41 (46%)	21 (23.5%)	10 (40%)	6 (24%)	9 (60%)	5 (33%)	4 (31%)	2 (15%)	9 (50%)	3 (16.6%)	9 (50%)	5 (27%)
nEV	10 (11%)	10 (11%)	3 (12%)	3 (12%)	3 (20%)	3 (20%)	1 (8%)	1 (8%)	0	0	3 (17%)	3 (17%)

*CR* complete response, *PR* partial response, *ORR* objective response rate, *DCR* disease control rate, *PD* progressive disease, *SD stable disease (more than 2 months with a confirmed response), *nEV* non-evaluable.

In the V trial, one patient (4%) had a CR (4%) and six (24%) experienced a PR, leading to an ORR of 28%. The V + P + C trial had one confirmed CR and two confirmed PRs, for a confirmed ORR of 20%. The V + E trial, V + C arm, and V + S arm had no confirmed CRs but had 4, 2, and 3 confirmed PRs, respectively, for confirmed ORRs of 22, 23, and 17%, respectively. The patients in the V + P + C trial and those in the V + S arm had the highest rates of SD (33 and 27%, respectively).

### Survival outcomes

**Fig. 2:**
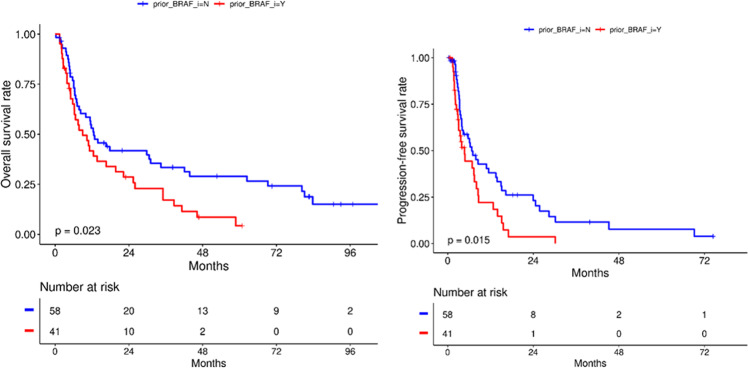
Overall survival and progression-free survival outcomes in the 4 phase 1 trials. No clinically significant impact on survival outcomes were seen when vemurafenib monotherapy was compared with combination regimens.

All 99 patients were included in the survival outcomes analysis (Fig. 2). Overall, the median OS duration was 11.5 months (range, 7.8–17.8 months), and the median progression-free survival (PFS) duration was 6.27 months (range, 3.90–8.50 months). Notably, no statistically significant association was noted among the four phase 1 trials and the patient outcomes, except for the inferior outcomes in the V + P + C trial and crossover patients. In the V trial, the median OS duration was 16.57 months and the median PFS duration was 8.5 months. In the V + C arm, the median OS duration was 3.5 months (*P* = 0.513; HR, 1.28; 95% CI 0.6–2.7) and the median PFS duration was 7.5 months (*P* = 0.0716; HR, 2.05; 95% CI, 0.93–4.5). In the V + E trial, the median OS duration was 8.9 months (*P* = 0.196; HR, 1.57; 95% CI, 0.78–3.15) and the median PFS duration was 3.7 months (*P* = 0.0936; HR, 1.8; 95% CI, 0.90–3.60). In the V + P + C trial, the median OS duration was 6.6 months and was statistically significant (*P* = 0.011; HR, 2.4; 95% CI, 1.22–4.7) and the median PFS was 4.9 months (*P* = 0.229; HR, 1.57; 95% CI, 0.75–3.29). Lastly, in the V + S arm, the median OS was 25.4 months (*P* = 0.589; HR, 1.193; 95% CI, 0.62–2.26) and the median PFS was 7.9 months (*P* = 0.672; HR, 1.16; 95% CI, 0.58–2.30).

**Fig. 3:**
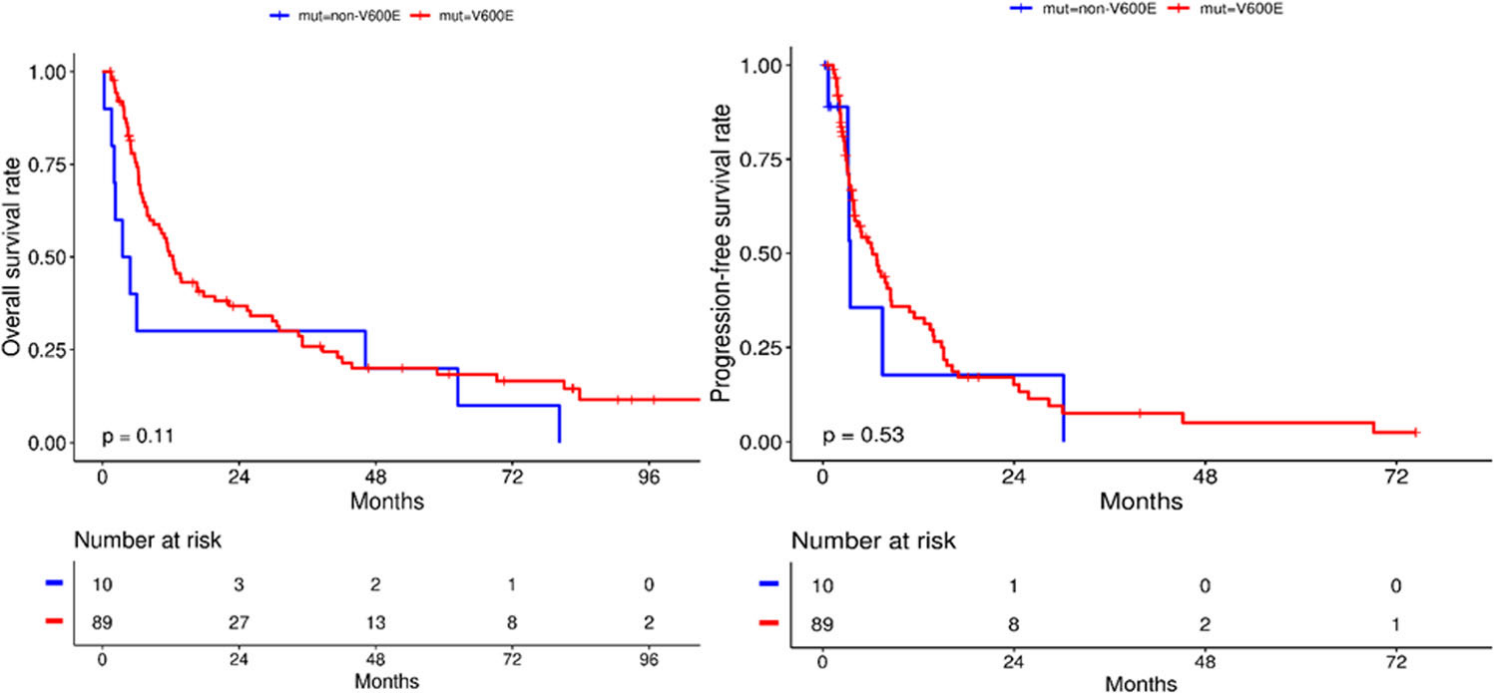
Survival outcomes based on prior BRAF therapy exposure. The median OS (12.6 months) was statistically significant in the BRAF therapy naïve group compared to the BRAF therapy refractory group (10.4 months) (*P* = 0.024). The median PFS was also statistically significant in the BRAF therapy naïve group (7 months) compared to the BRAF therapy refractory group (4.7 months) (*P* = 0.016).

We examined the role of rechallenge with vemurafenib in patients who were refractory to prior BRAF inhibitor therapy, as depicted in Fig. 3. 41% of patients had received prior BRAF-based therapies in the combination arms, while 58% of patients were BRAF therapy naive. The median OS was statistically significant at 12.6 months in the BRAF therapy naïve group compared to 10.4 months in the BRAF therapy refractory group (*P* = 0.024; HR, 1.69; 95% CI 1.07–2.68). The median PFS was also statistically significant between both groups, with 7 months in the BRAF therapy naïve group compared to 4.7 months in the BRAF therapy refractory group (*P* = 0.016; HR, 1.80; 95% CI 1.11–2.91).

**Fig. 4:**
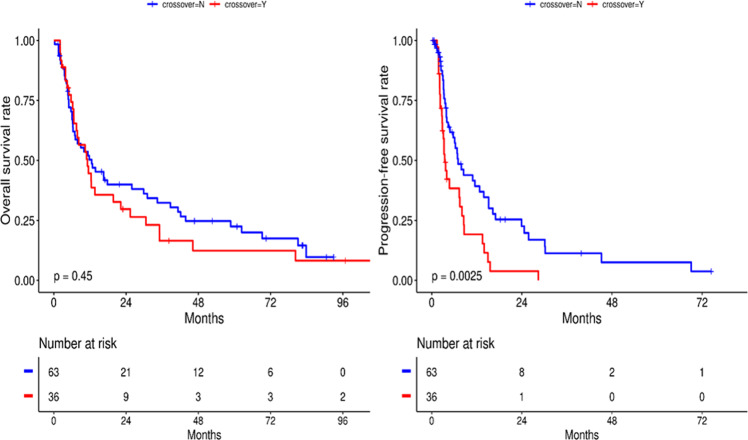
Survival outcomes based on BRAF V600E and non-V600E mutations. No statistical significance was seen between both groups, although the median OS was higher in the V600E group at 12.3 months compared to 4.8 months in the non-V600E group and median PFS was 6.8 months in the V600 group and 3.5 months in the non-V600E group.

Across all trials, ninety percent (*n* = 89) of patients had a BRAF V600E mutation and eleven percent (*n* = 10) of patients had non-BRAF V600E mutations. When we examined the survival outcomes among these two groups, no statistical significance was seen, although the median OS was higher in the V600E group at 12.3 months compared to 4.8 months in the non-V600E group (*P* = 0.10; HR, 0.57; 95% CI 0.29–1.13). The median PFS was 6.8 months in the V600 group and 3.5 months in the non-V600E group (*P* = 0.52; HR, 0.76; 95% CI 0.33–1.77) as seen in Fig. 4.

Stratification according to melanoma and non-melanoma tumors was conducted to prevent confounding variability in analysis. Notably, melanoma patients in the V + C arm had a median OS duration of 35 months (95% CI, 5.03-not applicable [N/A]) and a median PFS duration of 8.5 months (95% CI, 2.87-N/A). Melanoma patients in the V + S arm had a median OS duration of 19.7 months (95% CI, 10.87-N/A) and a median PFS duration of 6.8 months (95% CI, 2.07-N/A). Melanoma patients in the V + E trial had a median OS duration of 6 months (95% CI, 3.50-N/A) and a median PFS duration of 3.9 months (95% CI, 3.20-N/A). Non-melanoma patients in the V + S arm had a median OS duration of 30.5 months (95% CI, 7.83-N/A) and a median PFS duration of 17 months (95% CI, 6.27-N/A), whereas patients in the V + P + C trial had a shorter median OS duration of 2.2 months (95% CI, 1.60-N/A) and a shorter median PFS duration of 3 months (95% CI, 3.07-N/A), but these differences were not found to be significant.

**Fig. 5:**
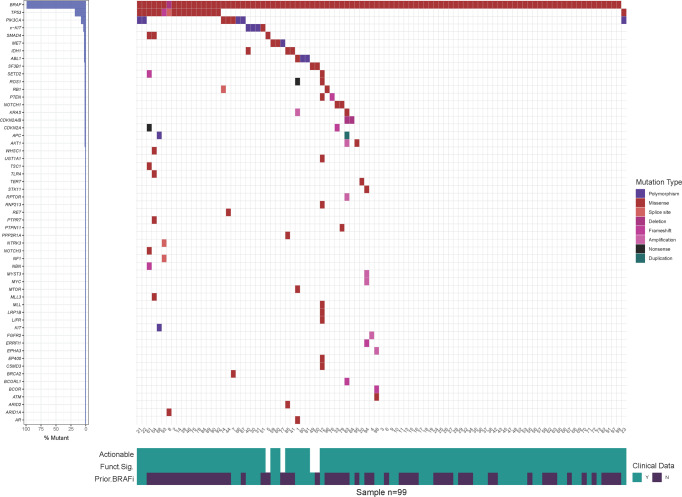
Survival outcomes based on crossover status. The median progression-free survival duration of crossover patients (3.67 months) was significantly shorter than that of non-crossover patients (7 months) (*P* = 0.0025).

Thirty-six patients (36%) crossed over to one or more of the above trials at the time of disease progression. Their median OS duration was 11.2 months (range, 7.5–25.4 months), and their median PFS duration was 3.67 months (range, 2.87–8.53 months) compared to non-crossover patients where median OS was 12.6 months (range, 7.0–31.1 months) and median PFS was 7 months (range, 5.7–15.17 months). Notably, the median PFS duration of the crossover patients was significantly shorter than that of the non-crossover patients (*P* = 0.0025; HR, 2.089; 95% CI, 1.2–3.4), whereas the median OS duration did not differ significantly between the groups (*P* = 0.45; HR, 1.192; 95% CI, 0.75–1.88) (Fig. 5). Our univariate analysis revealed a significant association between crossover status and PFS (*P* = 0.003; HR, 2.089; 95% CI, 1.283–3.402) but no association between age, gender, tumor type, or ethnicity and OS or PFS (Table 4).

**Table 4:** Univariate analysis of patient characteristics and survival outcomes.

Characteristic	OS HR (95% CI)	*P* value	PFS HR (95% CI)	*P* value
Age	1.004 (0.991–1.018)	0.56	0.998 (0.985–1.012)	0.8
Gender	1.056 (0.673–1.659)	0.81	0.863 (0.536–1.389)	0.54
Tumor type	0.753 (0.476–1.192)	0.23	0.638 (0.386–1.055)	0.08
Crossover status	1.192 (0.754–1.883)	0.45	2.089 (1.283–3.402)	0.003
Ethnicity				
White				
Asian	0.892 (0.123–6.469)	0.91	0.593 (0.082–4.305)	0.61
Black	0.420 (0.058–3.042)	0.39	1.417 (0.343–5.850)	0.63
Hispanic	0.737 (0.365–1.486)	0.39	0.720 (0.366–1.414)	0.34
Native American	7.818 (1.007–60.68)	0.049	5.7–6 (0.000-Inf)	0.99

*OS* overall survival, *HR* hazard ratio, *PFS* progression-free survival.

### Genomic landscape

**Fig. 6:**
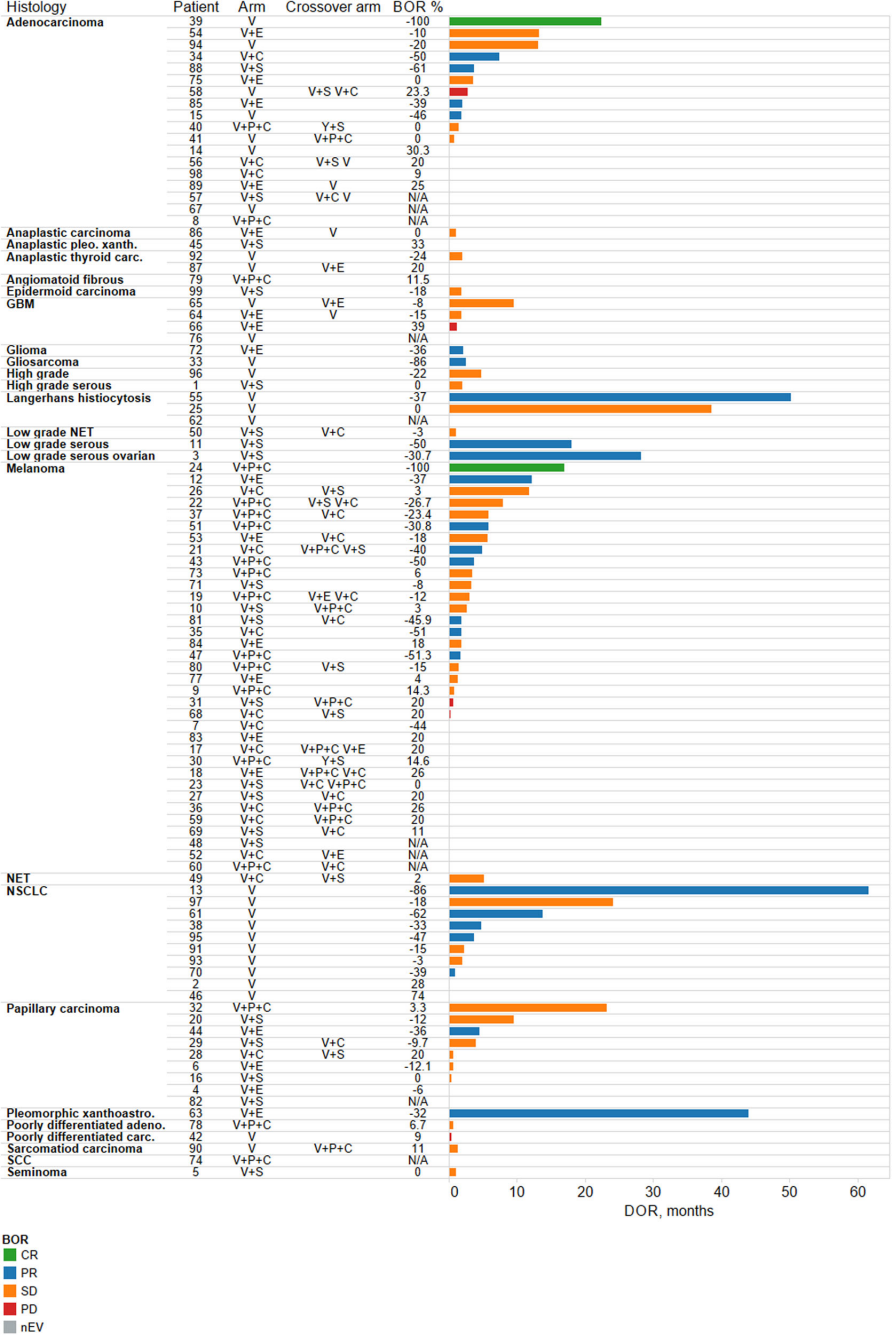
Integrative genomic landscapes of various solid tumors with genomic alterations in the pooled analysis of four phase one clinical trial. Each horizontal lane describes a single gene, the vertical lines represent different samples, and different colors indicate mutation types.

All 99 patients who participated in the four phase 1 V and combination trials had at least one genomic alteration, although in four patients, these alterations were not actionable or functionally significant. Ninety percent (*n* = 89) of patients had a *BRAF* V600E mutation and eleven percent (*n* = 10) of patients had *BRAF* V600K; R671Q; N581S; N486P 490del; D594G; K601Q; G469A; K601Q; M550I mutations, respectively. The most prevalent oncogenic driver mutations were in *BRAF* (49%), *TP53* (16%), *PIK3CA* (5%), *IDH1* (3%), *MET* (3%), *c-KIT* (3%), and others (21%). A genomic heat map of the somatic mutations frequently altered in the participants in the four trials is depicted in Fig. 6.

## Discussion

Results of this pooled analysis of four phase 1 trial with combinations of vemurafenib with cytotoxic chemotherapy or with *RAF*- or *mTOR*-targeting agents compared with vemurafenib monotherapy, do not significantly extend the OS or PFS of patients who have solid tumors with *BRAF* V600E mutations. Until 2011, no standard-of-care therapeutic agents had shown promise in improving clinical outcomes in patients with advanced melanoma. That year, vemurafenib became the first FDA-approved, targeted treatment developed through validated biomarker testing and illustrated the important role that precision oncology could play in the development of treatments for patients with *BRAF* V600E-mutated, advanced melanoma^21^. The phase 3 BRIM-3 trial showed that *BRAF*-targeted therapy had a rapid onset of action; tumor responses were seen by the first 2 months of therapy and occurred more rapidly than the responses to cytotoxic therapies. A robust 48% response rate to vemurafenib was achieved, but response durations were also relatively short (median, 5–10 months). Likewise, survival outcomes for vemurafenib were around 45 days for the PFS duration and 90 days for the OS duration leading to rapid progression^22,23^. In fact, almost half of the patients treated with vemurafenib experienced progression within 6 months. This problem led to the development of trametinib, cobimetinib, and binimetinib, which block *MEK1* and *MEK2* signals further down in the MAPK stream. The rationale behind the development of these drugs was to synergize and potentiate the downstream effects of *BRAF* inhibition via *MEK* inhibition and create a therapy with durable efficacy^24^. In the BRIM-7 trial, the combination of vemurafenib and cobimetinib elicited an impressive response rate of 87% and durable efficacy (mOS at 31 months; mPFS, 13.8 months). The median time to respond was 6 weeks, and response durations ranged from 2 to 6 months^25^.

As noted in the literature, resistance pathway mechanisms in *BRAF* V600E-positive tumors include the following: (1) *RAF* dimerization secondary to upregulation of wild-type *RAF* expression or *RAS* activity; (2) *BRAF* V600E splice mutations with deficient *RAS*-binding domains; and (3) Tyrosine kinase-facilitated stimulation of the *MAPK* pathway, which is vital in *MEK* inhibition^26^. Hence, this led to the exploration of combining *BRAF* and *MEK* inhibition for the treatment of tumors with *BRAF* V600E mutations. This led to the randomized phase 3 trial where dabrafenib and trametinib regimen was compared against dabrafenib in advanced melanoma with *BRAF* V600E or V600K mutation. The authors were able to demonstrate a 25% relative reduction in the risk of progression and a 37% relative reduction in the risk of death at the interim survival analysis with statistical significance with the combination regimen^24^. *BRAF* inhibition with or without *MEK* inhibition has since been shown to have activity against cancers other than melanoma, with the exception of colorectal cancer^5,17,27–31^. In addition to the upregulation of the *MAPK* pathway, several other mechanisms of *BRAF* inhibition resistance have been reported. These include the stimulation of yes-associated protein 1, extracellular signal-regulated kinase, platelet-derived growth factor receptor β (*PDGFRβ*), and mesenchymal-epithelial transition factor (*c-MET*), leading to conformal changes in the neuroblastoma *RAS* viral oncogene homolog (*NRAS*) gene with subsequent activation of phosphatidylinositol 3-kinase (*PI3K*) and the mechanistic target of rapamycin (*mTOR*) pathway^1,21,32^. BRAF aberrations have been reported as a mechanism of acquired resistance to different targeted therapies. Dagago-Jack et al. demonstrated how the addition of trametinib with osimertinib in EGFR-mutant NSCLC to target the acquired BRAF fusion resulted in a durable response for five months in the fifth line setting^33^. Although the above study assesses the evolution of genomic changes via tissue biopsy, liquid biopsy, such as cell-free DNA (cfDNA) analysis are feasible approaches in the current era for tracking resistance mechanisms of acquired resistance. These approaches can aid the results of this present study in designing biologically rational combinatorial approaches.

Given the historical success with combinations of BRAF and MEK inhibitors, the clinical trials NCT01524978, NCT01596140, NCT01636622, and NCT01531361, which we examined in this study, were designed to combine *BRAF* inhibition and the targeting of the *MAPK, mTOR,* and *MET* pathways to ensure synergistic cytotoxic suppression for heavily treated patients with BRAF mutations^17–20^. All four trials have since been published with varying confirmed rates of response (15–28%), with the best response rates seen in the vemurafenib monotherapy study. In that study, four patients with NSCLC, one with gliosarcoma, and one with ECD had PR lasting 3.7 to 61.5 months (for the NSCLC patients), 2.5 months, and 50.2 months, respectively and one patient with salivary adenocarcinoma had CR for 22 months. In the vemurafenib and paclitaxel and carboplatin trial, one patient with metastatic melanoma had a CR for 17 months, while two melanoma patients in the trial had PRs for 5.7 and 3.7 months, respectively. Notably, this trial had statistically significant inferior overall survival with a median OS duration of 6.6 months (*P* = 0.011; HR, 2.4; 95% CI, 1.22–4.7). Potential reasons include the inability to sustain BRAF inhibition in this combination, as 39% of patients required dose reduction and one death event occurred secondary to grade 4 thrombocytopenia. In the vemurafenib and crizotinib arm, one patient with melanoma and another with NSCLC had PRs for 4.9 months and 7.4 months, respectively. In the vemurafenib and everolimus trial, one patient with papillary thyroid carcinoma, one with melanoma, one with low-grade glioma, and one with optic glioma had PRs for 4.4. months, 12 months, 44 months, and 2 months, respectively. In the vemurafenib and sorafenib arm, two patients with ovarian cancer and one with NSCLC had PRs for 18, 28, and 3.7 months, respectively. These findings suggest that there may be value in pursuing the above novel combinations in various histologies. Prospective biomarker-driven exploratory studies are required to understand and select this subset of the patient population who may derive benefit from the above combinations.

Comparable safety data to prior published individual trials were seen in this study. Safety data from previous phase 3 trials indicate that the toxicity profile of BRAF and MEK inhibitors used in combination is similar to that of either agent used as monotherapy. In previous studies, compared with BRAF and MEK inhibition, vemurafenib monotherapy had a higher incidence of musculoskeletal side effects, which were mainly arthralgias and skin toxicities, including rashes and benign, precancerous, and cancerous lesions^22^. For the combination of vemurafenib and MEK inhibitors, the predominant adverse events included fever and gastrointestinal symptoms^25^. Similar results were seen in our study, in which around 32% of patients in the pooled analysis required dose reductions, primarily after treatment with combined agents rather than vemurafenib alone. However, the combination treatments were generally well-tolerated; only 7% of patients withdrew from the trials because of toxicity, and there was only one G5 adverse event.

Historically, it was thought that prior BRAF therapy exposure was a poor prognostic factor for survival among patients rechallenged with BRAF/MEK inhibitors. A phase 2 study from Belgium examined this issue in patients whose disease was resistant to immunotherapy and BRAF inhibitor–based therapy and who had a minimum of 12 weeks washout from their prior BRAF therapy. There were eight PRs, leading to a response rate of 32%, whereas BRIM-7 and other prospective studies that tested BRAF/MEK inhibition therapy in similar populations reported response rates ranging from 10 to 15%^25,34,35^. However, this finding did not translate in the current trials. In our study, approximately 36% of patients crossed over to one or more of the four respective phase 1 trials, but these patients had significantly shorter PFS than the non-crossover patient population. The reason for this result is unclear, but it may be attributable to a shorter washout time in crossover compared to non-crossover patients which could have led to the persistence of activated resistance pathways along with a heavily treated predisposition. Although 89% of patients in this pooled analysis had BRAF class 1 mutation as RAS-independent active monomers such as V600E, which is favored by vemurafenib for selective binding^36^, 11% of patients had class 2 mutations which are constitutively active RAS-independent dimers and class 3 mutations which have low/absent kinase activity where vemurafenib does not have activity^37^. Although the survival outcomes were not statistically significant, patients with BRAF V600E mutations had a 2–2.5-fold advantage in PFS and OS, which aligns with outcomes reported in literature^38^.

Multiple strides have been undertaken to find novel target combinations with vemurafenib beyond the pathways explored in our study. A strong rationale for the triple blockade of *BRAF, MEK*, and *EGFR* in *BRAF* V600E-positive advanced carcinoma of the colon and rectum was established after ref. ^39^ discovered that *EGFR* inhibition demonstrated synthetic lethality with *BRAF* V600E blockade. *EGFR* pathway is an adaptive mechanism of resistance in colorectal cancer and that concurrent *EGFR* pathway inhibition is needed in the treatment of *BRAF* V600-mutant colorectal cancer. In their preclinical studies, vemurafenib was combined with *EGFR* inhibitors like cetuximab and gefitinib and tested in colorectal cancer cell lines. The authors found that blocking the *BRAF* pathway stimulates the *EGFR* pathway, resulting in the poor efficacy of BRAF monotherapy^39^. Hence, the BEACON CRC trial combined encorafenib, cetuximab, and binimetinib and demonstrated that the combination was clinically efficacious, extended OS, and improved responses in patients with metastatic colorectal cancer and *BRAF* V600E mutations. The median OS was 9.0 months in the encorafenib, cetuximab, and binimetinib arm compared with 5.4 months in the standard-of-care arm (*P* < 0.001), and the confirmed ORR was 26% in the encorafenib, cetuximab, and binimetinib arm compared with 2% in the standard-of-care arm (*P* < 0.001)^40^.

Our study has some possible limitations. Because the trials investigated tumor-agnostic therapies, the enrolled patients had diverging disease characteristics, prior treatments, and performance status. Notably, a variety of treatment regimens were employed in the studies we examined; these regimens involved different drugs, doses, and durations of therapy. The sample sizes in each trial were small, and the studies were designed before the advent of immunotherapy for melanoma. Furthermore, these trials had long enrollment periods, in part due to interim approval of immunotherapy, the small number of patients with molecularly suitable tumors, and slow patient accrual for each study in a single institution. Most importantly, patients were not precisely matched to the combination regimens on the basis of their biomarkers, so there was no opportunity to explore the mechanistic impact of specific co-occurring alterations. Complicating this is also the fact that at the time of design of the above phase 1 trials, cfDNA, and comprehensive next-generation sequencing testing were not available to determine molecular pathways of acquired resistance. It should also be noted that the monotherapy and combination studies led to dose reductions in 28–50% of patients for tolerance. Thus it is difficult to draw definite conclusions surrounding efficacy and drivers of resistance.

Our findings suggest that, compared with vemurafenib monotherapy, combinations of vemurafenib with cytotoxic chemotherapy or with *RAF*- or *mTOR*-targeting agents do not significantly extend the OS or PFS of patients who have solid tumors with *BRAF* V600E mutations. Exploration of mechanistic translational molecular pathways that activate *BRAF* resistance and developing the means to therapeutically address them in this rapidly evolving landscape of precision oncology continues to remain vital. Gaining a better understanding of the molecular pathways involved in *BRAF* resistance will aid in designing sound, biologically efficient clinical trials with translational success.

## Methods

### Patient population

The research protocol was approved by The University of Texas MD Anderson Cancer Center’s Institutional Review Board. All patients provided written informed consent. The demographic, clinical, and histopathologic data of patients with BRAF V600-mutant solid tumors were pooled from 4 phase 1 dose-escalation trials (NCT01524978, NCT01596140, NCT01636622, and NCT01531361), which were conducted between January 2012 and October 2020^17–20^.

Any patient who had an advanced solid tumor refractory to standard-of care-treatment and a targetable *BRAF* mutation was invited to participate in the above trials. Patients underwent baseline dermatological exams, and any concerning skin lesions were addressed before trial participation. Qualified patients included those who had an ECOG performance status of two or less and suitable organ function as outlined in the originally published trials. The primary aims were to identify maximum-tolerated and recommended phase 2 therapy doses and DLTs and to establish the safety of the monotherapy and combination therapies. The secondary aim of the trials was to explore the antitumor potency of the tested drugs.

### Trial designs and schedules

The studies were open-label, non-randomized, phase 1 dose-escalation trials with a modified 3 + 3 design to explore safety by the recruitment of additional trial candidates.

In the vemurafenib monotherapy trial (NCT01524978), vemurafenib was administered as a single agent at an oral dose of 960 mg twice daily. In a sub-arm of the above trial, colorectal cancer patients received 960 mg of vemurafenib twice per day and cetuximab at a 400 mg/m^2^ loading dose and then received 250 mg/m^2^ intravenous cetuximab every week.

In the vemurafenib and paclitaxel and carboplatin trial (NCT01636622), 480 to 720 mg vemurafenib was administered orally twice per day for 21 days. Additionally, patients received 100 to 135 mg/m^2^ paclitaxel and carboplatin at a target area under the curve of 5–6 mg/mL/min given on the first day of each cycle.

In the vemurafenib and everolimus trial (NCT01596140), 720 mg vemurafenib and 5 mg everolimus were administered orally every day. Every cycle lasted 28 days. Two pediatric patients recruited after the dose-escalation phase were treated with 480 mg vemurafenib and 2.5 mg everolimus per day.

In the trial of vemurafenib with either crizotinib or sorafenib (NCT01531361), all patients received 240 to 960 mg vemurafenib orally twice per day for 28 days. Those in the crizotinib arm also received 250 mg of oral crizotinib twice per day, and those in the sorafenib arm also received 200 to 400 mg of oral sorafenib twice per day for 28 days.

### Trial assessments

Patients who experienced any clinical G3 or four nonhematologic toxicity, as defined in the National Cancer Institute Common Terminology Criteria for Adverse Events, version 4 (NCI-CTCAE v4.0)secondary to the investigational drug or combination; any G4 hematologic toxicity lasting 3 or more weeks (as defined by the NCI-CTCAE v4.0) or associated with bleeding and/or sepsis; any G4 nausea or vomiting lasting more than 5 days despite the maximum administration of anti-nausea regimens; or any severe or life-threatening complication or abnormality not defined in the NCI-CTCAE v4.0 that was attributable to the therapy observed during the first cycle were deemed to have DLTs. The maximum-tolerated dose was defined as the highest dose for which the DLT incidence was less than 33%. Computed tomography or magnetic resonance imaging was performed for baseline assessments and every 8 weeks to assess response. Antitumor responses were assessed using the response evaluation criteria in solid tumors (version 1.1).

### Statistical analysis

Demographic and clinical characteristics were analyzed using descriptive statistics. PFS was defined as the time from the first day of cycle 1 to the date of progression or death, whichever came first. Patients who were alive and progression-free at the last clinical follow-up were censored at the date of the last clinical follow-up. OS was defined as the time from the first day of cycle 1 to death from any cause. Patients alive at the last follow-up were censored at the date of the last contact. Survival (PFS and OS) was analyzed using the Kaplan–Meier method from the time of trial participation and included median survivals (with 95% CIs). HRs and corresponding CIs and *P* values were computed using a Cox proportional hazards regression analysis. Clopper–Pearson exact binomial CIs were provided for estimates of proportions. Survival differences between treatment cohorts were assessed through the log-rank test with univariate analysis. All tests were two-sided, and *P* values < 0.05 were considered statistically significant. All statistical analyses were performed using R software, v3.6.0.

### Genomic analysis

Archived tumor specimens were analyzed at institutional Clinical Laboratory Improvement Amendments (CLIA)-certified laboratories for next-generation sequencing data. Data were reviewed using other platforms such as NeoGenomics, Aliso Viejo, CA, USA; Guardant360; Guardant Health, Redwood City, CA; and Foundation Medicine, Cambridge, MA, USA.

### Reporting summary

Further information on research design is available in the Nature Research Reporting Summary linked to this article.

## Supplementary information

REPORTING SUMMARY

## Data Availability

Trial data from the four phase 1 trials (NCT01524978, NCT01596140, NCT01636622, and NCT01531361) that have been analyzed retrospectively for this study is publicly available and published as quoted in refs. ^17–20^.
