# Supplementary material for: BRAF v600E–mutant cancers treated with vemurafenib alone or in combination with everolimus, sorafenib, or crizotinib or with paclitaxel and carboplatin (VEM-PLUS) study
Source: NPJ Precis Oncol. 2023 Feb 18;7:19. doi: 10.1038/s41698-022-00341-0 (PMC9938883; doi:10.1038/s41698-022-00341-0)
Supplement: Supplementary file 1 — REPORTING SUMMARY [file 41698_2022_341_MOESM1_ESM.pdf]

nature portfolio

Corresponding author(s): Vivek Subbiah MD

Last updated by author(s): 2022/10/10

Reporting Summary

Nature Portfolio wishes to improve the reproducibility of the work that we publish. This form provides structure for consistency and transparency in reporting. For further information on Nature Portfolio policies, see our Editorial Policies and the Editorial Policy Checklist.

Statistics

For all statistical analyses, confirm that the following items are present in the figure legend, table legend, main text, or Methods section.

n/a	Confirmed	
☐	☒	The exact sample size (n) for each experimental group/condition, given as a discrete number and unit of measurement
☒	☐	A statement on whether measurements were taken from distinct samples or whether the same sample was measured repeatedly
☐	☒	The statistical test(s) used AND whether they are one- or two-sided<br>*Only common tests should be described solely by name; describe more complex techniques in the Methods section.*
☐	☒	A description of all covariates tested
☒	☐	A description of any assumptions or corrections, such as tests of normality and adjustment for multiple comparisons
☐	☒	A full description of the statistical parameters including central tendency (e.g. means) or other basic estimates (e.g. regression coefficient) AND variation (e.g. standard deviation) or associated estimates of uncertainty (e.g. confidence intervals)
☐	☒	For null hypothesis testing, the test statistic (e.g. F, t, r) with confidence intervals, effect sizes, degrees of freedom and P value noted<br>*Give P values as exact values whenever suitable.*
☒	☐	For Bayesian analysis, information on the choice of priors and Markov chain Monte Carlo settings
☒	☐	For hierarchical and complex designs, identification of the appropriate level for tests and full reporting of outcomes
☒	☐	Estimates of effect sizes (e.g. Cohen's d, Pearson's r), indicating how they were calculated

Our web collection on statistics for biologists contains articles on many of the points above.

Software and code

Policy information about availability of computer code

Data collection	No software was used for data collection
Data analysis	Demographic and clinical characteristics were analyzed using descriptive statistics. Survival (PFS and OS) was analyzed using the Kaplan-Meier method from the time of trial participation and included median survivals (with 95% CIs). HRs and corresponding CIs and P values were computed using a Cox proportional hazards regression analysis. Clopper-Pearson exact binomial CIs were provided for estimates of proportions. Survival differences between treatment cohorts were assessed through the log-rank test with univariate analysis. All tests were 2-sided, and P values < .05 were considered statistically significant. All statistical analyses were performed using R software, v3.6.0.

For manuscripts utilizing custom algorithms or software that are central to the research but not yet described in published literature, software must be made available to editors and reviewers. We strongly encourage code deposition in a community repository (e.g. GitHub). See the Nature Portfolio guidelines for submitting code & software for further information.

Data

Policy information about availability of data

All manuscripts must include a data availability statement. This statement should provide the following information, where applicable:

- Accession codes, unique identifiers, or web links for publicly available datasets
- A description of any restrictions on data availability
- For clinical datasets or third party data, please ensure that the statement adheres to our policy

All data that is available is reported in the paper. Since this was a retrospective review data and pooled analyses from many Phase 1 trials there is no current plan for data sharing.

Human research participants

Policy information about studies involving human research participants and Sex and Gender in Research.

Reporting on sex and gender	Ninety-nine patients (57 males and 42 females) with BRAF mutant solid tumors were enrolled across four phase I clinical trials
Population characteristics	Age, sex, number of prior therapies, type of tumor, prior BRAF therapy and cross-over patients
Recruitment	Patients who were recruited at UT MD Anderson Cancer Center through clinic visits were retrospectively reviewed. The recruitment was based on physician's decision, which may have lead to selection bias.
Ethics oversight	The research protocol was approved by The University of Texas MD Anderson Cancer Center's Institutional Review Board.

Note that full information on the approval of the study protocol must also be provided in the manuscript.

Field-specific reporting

Please select the one below that is the best fit for your research. If you are not sure, read the appropriate sections before making your selection.

☒ Life sciences ☐ Behavioural & social sciences ☐ Ecological, evolutionary & environmental sciences

For a reference copy of the document with all sections, see nature.com/documents/nr-reporting-summary-flat.pdf

Life sciences study design

All studies must disclose on these points even when the disclosure is negative.

Sample size	Sample size was based on number of patients enrolled in the four phase 1 trials during the specified time period. Sample size calculations were performed on each of the published trial separately.
Data exclusions	No data was excluded in this pooled analysis.
Replication	Replication was not feasible in this study. This is a pooled analysis study among patients enrolled in the four phase 1 trials.
Randomization	No randomization was conducted due to this retrospective research.
Blinding	No blinding was conducted due to the retrospective research.

Reporting for specific materials, systems and methods

We require information from authors about some types of materials, experimental systems and methods used in many studies. Here, indicate whether each material, system or method listed is relevant to your study. If you are not sure if a list item applies to your research, read the appropriate section before selecting a response.

Materials & experimental systems

n/a	Involved in the study
☒	☐ Antibodies
☒	☐ Eukaryotic cell lines
☒	☐ Palaeontology and archaeology
☒	☐ Animals and other organisms
☐	☒ Clinical data
☒	☐ Dual use research of concern

Methods

n/a	Involved in the study
☒	☐ ChIP-seq
☒	☐ Flow cytometry
☒	☐ MRI-based neuroimaging

Clinical data

Policy information about clinical studies

All manuscripts should comply with the ICMJE guidelines for publication of clinical research and a completed CONSORT checklist must be included with all submissions.

Clinical trial registration	https://clinicaltrials.gov/ct2/show/NCT01531361; https://clinicaltrials.gov/ct2/show/NCT01636622; https://clinicaltrials.gov/ct2/show/NCT01596140; https://www.clinicaltrials.gov/ct2/show/NCT01524978
Study protocol	NCT01524978, NCT01596140, NCT01636622, and NCT01531361
Data collection	Data was collected at UT MD Anderson Cancer Center
Outcomes	Clinical outcomes (progression-free survival, overall survival and clinical response) that were not predefined were assessed using logistic regression and Kaplan-Meier analysis. Due to the nature of this observation study, there are no predefined primary and secondary endpoints.
